# Dihydroartemisinin Ameliorates Learning and Memory in Alzheimer’s Disease Through Promoting Autophagosome-Lysosome Fusion and Autolysosomal Degradation for Aβ Clearance

**DOI:** 10.3389/fnagi.2020.00047

**Published:** 2020-03-02

**Authors:** Yueyang Zhao, Zhimin Long, Ya Ding, Tingting Jiang, Jiajun Liu, Yimin Li, Yuanjie Liu, Xuehua Peng, Kejian Wang, Min Feng, Guiqiong He

**Affiliations:** ^1^Neuroscience Research Center, Chongqing Medical University, Chongqing, China; ^2^Department of Human Anatomy, Basic Medical School, Chongqing Medical University, Chongqing, China; ^3^Wuhan Children’s Hospital, Tongji Medical College, Huazhong University of Science & Technology, Wuhan, China

**Keywords:** Alzheimer’s disease, amyloid-beta, dihydroartemisinin, autophagic flux, autolysosomal degradation, autophagosome-lysosome fusion, cognitive deficits

## Abstract

Dihydroartemisinin (DHA) is an active metabolite of sesquiterpene trioxane lactone extracted from Artemisia annua, which is used to treat malaria worldwide. DHA can activate autophagy, which is the main mechanism to remove the damaged cell components and recover the harmful or useless substances from eukaryotic cells and maintain cell viability through the autophagy lysosomal degradation system. Autophagy activation and autophagy flux correction are playing an important neuroprotective role in the central nervous system, as they accelerate the removal of toxic protein aggregates intracellularly and extracellularly to prevent neurodegenerative processes, such as Alzheimer’s disease (AD). In this study, we explored whether this mechanism can mediate the neuroprotective effect of DHA on the AD model *in vitro* and *in vivo*. Three months of DHA treatment improved the memory and cognitive impairment, reduced the deposition of amyloid β plaque, reduced the levels of Aβ40 and Aβ42, and ameliorated excessive neuron apoptosis in APP/PS1 mice brain. In addition, DHA treatment increased the level of LC3 II/I and decreased the expression of p62. After Bafilomycin A1 and Chloroquine (CQ) blocked the fusion of autophagy and lysosome, as well as the degradation of autolysosomes (ALs), DHA treatment increased the level of LC3 II/I and decreased the expression of p62. These results suggest that DHA treatment can correct autophagic flux, improve autophagy dysfunction, inhibit abnormal death of neurons, promote the clearance of amyloid-β peptide (Aβ) fibrils, and have a multi-target effect on the neuropathological process, memory and cognitive deficits of AD.

## Introduction

Alzheimer’s disease (AD) has been known as one of the neurodegenerative diseases, manifested by chronic and progressive memory and cognitive deficits (Luheshi et al., 2008). Histopathologically, AD manifests *via* synaptic abnormalities, neuronal degeneration as well as the deposition of extracellular amyloid plaques and intraneuronal neurofibrillary tangles, which lead to a decline in memory and other cognitive functions. Beta-amyloid peptide (Aβ), which is mainly produced by the abnormal shearing of amyloid precursor protein (APP) by β-secretase and γ-secretase, respectively, is the core of senile plaque (SP) and plays a key role in AD pathogenesis (Folch et al., 2018). Excessive production and degradation of Aβ in cells lead to an imbalance and accumulation of Aβ metabolism. The imbalance of Aβ metabolism further leads to form extracellular SPs and a series of pathological changes of AD. Aβ excessive formation and blocked degradation impel SP deposition in the brain that, subsequently, accelerate the occurrence of overactive microglia, excessive apoptotic neurons and cerebral atrophy (Selkoe and Hardy, 2016; Nixon, 2017). In recent years, it has been found that extracellular Aβ is only the result of its toxic effect on cells, and the accumulation of intracellular Aβ is the fundamental factor leading to the cytotoxic effects (Esquerda-Canals et al., 2017). The degradation of extracellular Aβ is mainly completed by the insulin-degrading enzyme (IDE) and enkephalinase (Yamamoto et al., 2013, 2014), while the degradation of intracellular Aβ is mainly transported to lysosome through endocytosis or autophagy (Thal, 2015). The autophagy-lysosomal system dysfunction could directly affect APP metabolism and promote β-amyloidogenesis (Nixon, 2017). Thus, maintaining the stability of the autophagy–lysosomal network and strengthening the degradation of Aβ peptides in the lysosomes digestion system might be a promising strategy for treating AD. Macroautophagy (hereafter referred to as autophagy) is the main form of autophagy, and its role in neurodegenerative diseases has attracted much attention. Autophagy in healthy neurons is characterized by its high efficiency and continuous activation but low level (Boland et al., 2008) that accelerates the clearance of toxic and damaged intraneuronal and extracellular protein aggregates in lysosomes digestion system (Fecto et al., 2014). While in AD, autophagosomes (APs) accumulate owing to the stimulation of initiation of autophagy and sluggish rate of APs formation associated with failure to achieve adequate lysosome fusion as well as digestion (Uddin et al., 2019). Autophagy increases in the early stage of AD, which can play a role in scavenging Aβ, but excessive autophagy itself can also lead to increased production of Aβ. With the development of AD, the clearance of Aβ decreased due to the fusion of APs and lysosomes. The accumulation of APs due to the inhibition of degradation led to the further increase of Aβ production (De Strooper and Karran, 2016; Esquerda-Canals et al., 2017). So it has been reported that autophagy acts as a “double-edged sword” in the development of AD (Martinet et al., 2009; Choi et al., 2018; Hamano et al., 2018).

Autophagy flux, the rate at which long-lived protein aggregates are degraded by autophagy (Klionsky et al., 2016), includes the whole autophagy process, including the formation of autophagy structure, the transport of substrate to lysosome, the degradation of substrate and the release of macromolecule substances back to the cytoplasm (Yoon and Kim, 2016). In AD, dysfunctional autophagic flux was characterized as accumulating levels of APs in dystrophic neurites, whereas APP, Aβ peptides, β-site APP cleaving enzyme (BACE1) protein, damaged mitochondria and Golgi fragments are rich and cannot be cleared away by endosomal–lysosomal degradation process (Yu et al., 2005; Joshi and Wang, 2015; Feng et al., 2017; Kerr et al., 2017; Nixon, 2017). Therefore, the accumulation of APs not only affects the clearance of Aβ in cells but also could be the place where Aβ is produced. With increasing clinical researches and observations on the etiology, pathogenesis and candidate drugs of AD, the drugs which can reduce the production of pathological Aβ and promote the degradation of Aβ will be a new direction for the development of anti-AD drugs. Thus, maintaining highly efficient autophagy flux in brain homeostasis for maintaining Aβ-related production and degradation balance might be a promising strategy to treat AD.

In recent years, Chinese herbal medicine has received great attention in maintaining the efficient flux of autophagy in the brain. Artemisinins, a class of sesquiterpene trioxane lactone agents extracted from the ancient Chinese *herb Artemisia annua L*, has been used effectively to treat malaria (Lam et al., 2018) with universally acknowledged safety records in clinical trials (Kloprogge et al., 2018; Lohy Das et al., 2018). The main mechanism by which artemisinin acts in the treatment of malaria is activating autophagy to change the membrane structure of the parasite and, subsequently, starving the parasite (Chen et al., 2000). However, whether artemisinin can treat AD by regulating autophagy or not remains unclear. Dihydroartemisinin (DHA), the active metabolite of artemisinin, exhibits an ample array of autophagic activities as a drug intervention in many diseases (Jia et al., 2014; Jiang et al., 2016; Zhang et al., 2017), but no such study of DHA for AD treatment has ever been reported. One research has shown that DHA could be liable to penetrate the brain-blood barrier (Xie et al., 2009). In addition, low doses of DHA have beneficial effects on brain diseases, for example, experimental cerebral malaria in mice (Dormoi et al., 2013). In the present study, we used DHA to treat APP/PS1 double transgenic AD mice and AD model cells, intending to investigate whether DHA would maintain Aβ related production and degradation balance *via* promoting autophagy flux and exert a protective effect in AD. *In vitro* and *in vivo* results suggested that DHA alleviated memory deficits, decreased Aβ production and neuritic plaque formation, ameliorates the autophagy flux in the brains of AD mice. The multitarget-regulating effects of DHA on autophagic flux were verified in AD cell models pretreated with chloroquine (CQ) and bafilomycin A1. In brief, our data suggest that DHA is effective in the prevention and treatment of AD through promoting autophagosome-lysosome fusion and autolysosomal degradation in autophagic flux to clear Aβ.

## Materials and Methods

### Chemicals

**Figure 1:**
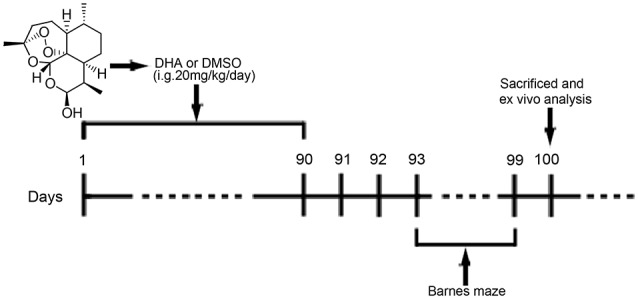
Chemical structure of dihydroartemisinin (DHA) and experimental protocol scheme. Mice received intragastric (i.g.) treatments for 90 days until the beginning of the behavioral tests. From the 93rd day of the experimental protocol, the animals were subjected to Barnes maze tests. On the 100th day, the mice were euthanized for *in vivo* analysis.

DHA (≥98%, Aladdin, Shanghai, China; Figure 1) was prepared and characterized in our laboratory according to previously described methods (Peters et al., 2003). DHA was dissolved in 10% dimethyl sulfoxide (DMSO, Amresco, USA) for oral treatment of mice. For cell treatments, DHA, bafilomycin A1 (Selleck, USA) and rapamycin (Gene Operation, USA) were dissolved in 100% DMSO as 50 mM stock solutions and stored for later use. CQ was dissolved in phosphate-buffered saline (PBS; 1× pH 7.4) as a 50 mM stock solution.

### Animals and Cells

In our pre-experiments, five male mice and five female mice per group were taken to assess their behavioral and pathological changes in the AD pathological course. We preliminarily found that no significant difference can be noted in DHA-treated model mice across genders. Thus, we chose only male mice for this study. The experiments were carried out utilizing male APPswe/PSEN1dE9 double-transgenic (APP/PS1) mice and non-transgenic (wild type, WT) littermates that were purchased from the Biomedical Research Institute of Nanjing University (Nanjing, China). A number of qualitative qualification for mice: 201803104 and 201806178. The mice were housed from ages 3- to 9-months-old in a laminar flow rack exposed to a 12 h light/dark cycle, with free access to water and standard rodent chow. All experimental procedures were performed in accordance with the guidelines approved by the Animal Protection and Ethics Committee of Chongqing Medical University.

Mouse neuroblastoma cell line Neuron-2a/APPswe (N2a-APP) cells and differentiated SH-SY5Y cells stably transfected with the APPswe gene (APP-SH-SY5Y) were gifts from Professor Zhifang Dong at the Laboratory of Translational Medical Research in Cognitive Development and Learning and Memory Disorders, Children’s Hospital of Chongqing Medical University (Chongqing, China), and the APP-SH-SY5Y cells were confirmed to be human through STR profiling. The N2A-APP cells were maintained in 90% DMEM (Gibco/Thermo Fisher Scientific, Waltham, MA, USA) and 10% fetal bovine serum (FBS, Biological Industries, USA) with 100 μg/ml G418. The APP-SH-SY5Y cells were cultured in 90% DMEM with 10% FBS, 100 U/ml penicillin and 100 μg/ml streptomycin. All cells were maintained at 37°C in 5% CO_2_ and 95% air (v/v).

### Drug Treatment/Administration

At 6 months old, APP/PS1 mice and WT mice were randomly divided into the DHA-treated AD group, DMSO-treated AD group, and DMSO-treated WT group (15 per group) and were administered DHA (20 mg/kg/day, intragastric administration, once daily) or an equal amount of DMSO for 3 months. Pharmacological studies using a dosage of 20 mg/kg/day per mouse were based on the scope of safe doses for adults, children and pregnant women from related studies (Chairat et al., 2018; Kloprogge et al., 2018; Chotsiri et al., 2019) and the formulas in the Conversion of Animal Doses to Human Equivalent Doses FDA guidelines (U.S. Department of Health and Human Services, Center for Drug Evaluation and Research (CDER), 2005). In these studies, 3 months of treatment for 6-month-old APP/PS1 mice were in accordance with the initial stage of Aβ deposition in this mouse model Xiang et al., 2015), which might support the preventive potential of DHA treatment in AD. After 3 months of treatment, behavioral tests were initiated. The day after the final day of behavioral tests, the mice were euthanized, and the brains were removed for *ex vivo* experiments. The experimental protocol is shown in Figure 1. Because artemisinin and its derivatives were mainly metabolized in the liver (Gautam et al., 2009), the safety of DHA in mice for three months was reflected by the concentration of the serum GPT/ALT and GOT/AST B36 (Supplementary Figure S1) and was verified it drug safety.

Bafilomycin A1 was diluted to 1 nM for APP-SH-SY5Y cells and 50 nM for N2A-APP cells (Guo et al., 2017). CQ was diluted to 10 μM for APP-SH-SY5Y cells and 25 μM for N2A-APP cells (Xu et al., 2018). Rapamycin was diluted to 100 nM for APP-SH-SY5Y cells and N2A-APP cells (Xu et al., 2018). The final concentration of DMSO was <0.1%. The CON groups were treated with the same concentration of DMSO or PBS.

### Cell Proliferation Assay

The cells were incubated in 96-well flat-bottom plates (1.0 × 10^4^ cells/well) with 0, 0.05, 0.5, 1, 5 and 50 μM DHA for 24 h before cell proliferation was assessed. Cell growth was analyzed using CCK-8 cell proliferation and cytotoxicity assay kit (Solarbio, Shanghai, China). Absorbance at 450 nm was measured using an ultra microplate reader (Thermo Scientific, USA).

### Behavioral Tests

Spatial learning and memory were assessed using the Barnes maze task in accordance with Pompl et al. (1999) but with minor modifications (Souza et al., 2012). The Barnes maze consists of a flat, circular disk (122 cm diameter) with 18 circular holes (5 cm diameter) at equal distances around the perimeter and rising 100 cm above the floor. The escape box (13 × 29 × 14 cm) was kept under one hole. The mice learned the location of the escape box under the hole using spatial reference points fixed to the wall. Animals were trained in the Barnes maze on the first day for one trial. Training consisted of placing the animal in a black box and leaving it for a minute. Then, the black box was placed in the center of the Barnes maze. The black box was removed, and then the training began. The mice freely explored the maze to find the escape box under the hole. The maximum latency to finding the escape box was 300 s. The latency to reach the escape box and the number of wrong holes was measured. After the first day of training, the test began, and the data for two trials were recorded over the next 5 days. On the seventh day, the escape box was removed, each mouse was placed in the center of the maze. The number of attempts to find the escape box and the number of other holes was reported.

### Preparation of Brain Tissue and Cells

At the end of treatment, the mice were euthanized by CO_2_ inhalation. Mice (*n* = 16 per group) were perfused transcardially with 0.01 M PBS (pH 7.4). The left cerebral hemisphere was bluntly dissected to the hippocampus and cortex for ELISA, and the right cerebral hemisphere was prepared for histological staining and Western blotting (WB). The rest cerebral hemisphere was used for transmission electron microscopy (TEM).

For histological staining, excised brains were immersed in 4% paraformaldehyde overnight, 20% sucrose for 24 h and 30% sucrose for another 48 h. Then, the brains were embedded in optimum cutting temperature compound in a freezing microtome, and 10 μm-thick sections were cut with a freezing microtome (Leica, Germany).

For TEM, mice from each group were transcardially perfused with 0.01 M PBS, followed by 2.5% glutaraldehyde 4% paraformaldehyde in 0.01 M PBS. The brain was quickly stripped in an ice bath. Brain tissues (1 mm^3^ thick) were cut from the hippocampal CA1 area and were fixed in 2.5% special glutaraldehyde solution for 2 h for TEM, washed several times with 0.01 M PBS, postfixed in 1% osmium tetroxide in 0.01 M PBS for 2 h and dehydrated with gradient alcohol. Tissue samples were embedded in Epon812 epoxy resin. Tissue blocks were then cut into 1-μm sections, placed on slides, stained with azure-methylene blue (Sino Chemical Co., Ltd., Zhengzhou, China), and visualized under a light microscope (Leica Microsystems, Wetzlar, Germany). The areas were selected from semi-thin sections and then cut into thin sections. The APP-N2a cells were prefixed in a 2.5% glutaraldehyde solution overnight at 4°C and postfixed in cold 1% aqueous osmium tetroxide for 1 h at 4°C. The samples were rinsed three times with PBS, dehydrated in a graded series of 25%–100% ethanol, embedded in fresh resin and polymerized at 60°C for 24 h. The samples were sectioned on a Leica EM UC6 ultramicrotome at 60–80 nm and collected on pioloform-coated Cu2*1 oval slot grids (Electron Microscopy Sciences, Hatfield, PA, USA).

### TEM

After uranyl acetate/lead citrate double staining (Sansd Plastic Co., Ltd, Fujian, China), neurons, gliocytes and their ultrastructures in brain sections were observed by TEM (Philips, Amsterdam, Netherlands). For cells, the sections were subsequently examined under a Hitachi7500 transmission electron microscope (Philips, Amsterdam, Netherlands). The morphological ultrastructures and status of APs and lysosomes in cells were photographed for each group.

### Histological Staining

For Thioflavin S staining, brain slices were mounted onto slides and washed three times with 0.01 M PBS for 5 min each. The washed brain slices were incubated in acetone for 10 min at room temperature, washed in 80% ethanol, 70% ethanol, and distilled water for 30 s, respectively. After that, slices were incubated in 0.1% KMnO_4_ for 30 s and then washed with distilled water, 70% ethanol, and 80% ethanol for 30 s. Subsequently, Thioflavin S (0.1% in 80% ethanol) was dripped on the slides for 15 min at room temperature in the dark. Finally, the sections were covered with coverslips. They were then photographed using fluorescence microscopy.

For immunohistochemistry, brain sections were mounted onto slides for staining. The slices were incubated in 88% formic acid for 10 min and washed in 0.01 M PBS three times. Then, the slices were incubated in 3% H_2_0_2_ peroxidase and citrate buffer (Bioss, Beijing, China; pH 6.0) for 30 min. Tissues were then blocked with 5% fetal calf serum (HyClone, Logan, Utah, UT, USA) in 0.3% Triton X-100 for 30 min at 37°C. Then, the tissues were incubated overnight at 4°C with the mouse monoclonal antibody 4G8 (1:200 dilution; Biolegend, USA). After rinsing, a biotinylated secondary antibody (1:200 dilution; Vector Laboratories, Burlingame, CA, USA) was added to tissue sections for 30 minutes at 37°C, followed by the avidin-biotin-peroxidase complex (Vectastain ABC kit; Vector Laboratories) according to the manufacturer’s protocol. Immunoreactivity was visualized. Plaques were visualized by the ABC and diaminobenzidine (DAB) method and counted under microscopy at 40× magnification. A minimum of three washes with 0.01 M PBS was completed between steps.

To assess autophagic flux in response to DHA, autophagic agonists and antagonists were used, and a tandem mRFP-GFP-LC3 adenovirus (Hanheng Biotechnology Co Ltd., Shanghai, China) was transfected into cultured APP-SH-SY5Y cells for 24 h at an MOI of 50. On the 2nd day following transfection, 1 μM DHA, 100 nM rapamycin, 10 μM CQ, and 1 nM bafilomycin A1 were added to the corresponding groups for 24 h. After fixing the cells with PFA and DAPI staining, autophagy was observed under a TCS-TIV confocal laser scanning microscope (Leica Microsystems). The tandem mRFP-GFP-LC3 protein showed both red (mRFP) and green (GFP) fluorescence at neutral pH and formed yellow (red+green) puncta that represent APs formation (Kimura et al., 2007). The relative ratio of positive dots near the nucleus vs. the total number of dots is an index of autophagic flux.

### Western Blotting Analysis

Protein expression was analyzed by immunoblot analysis. Total protein extract was prepared using RIPA lysis buffer (Beyotime Biotechnology, Shanghai, China), which was supplemented with phenylmethanesulfonylfluoride (Beyotime Biotechnology, Shanghai, China) according to the manufacturer’s instructions. Total protein concentration was determined using an enhanced BCA protein assay kit (Beyotime Biotechnology, Shanghai, China). Equal amounts of proteins were resolved using a 6%–12% SDS-PAGE gel kit (CWBIO, Beijing, China) and transferred onto polyvinylidene fluoride membranes (Millipore, USA). The membranes were incubated overnight at 4°C with the following primary antibodies: SQSTM1/p62 (#5114), LC3 (#3868), Rab7 (#9367), Cathepsin B/CTSB (#31718), ATG5 (DF6010), ATG12 (DF7937), ATG16L1 (DF3825), mammalian target of rapamycin (mTOR; AF6308), phospho-mTOR (Ser2448; AF3308), ULK1 (DF7588), phospho-ATG14 (Ser29; AF2320; all from Affinity Biosciences, USA); ATG14 (NBP2-36445, Novus Bio, USA); RILP (ab140188, Abcam, USA); Beclin1 (ab62557), Lamp1 (ab24170), APP (ab32136), BACE1 (ab183612), Presenilin 1/PS1 (ab76083), IDE (ab133561) and neprilysin/NEP (ab58968). After washing, the membranes were incubated for an hour at room temperature with horseradish peroxidase-conjugated secondary antibodies. The immunoblots were visualized using enhanced chemiluminescence WB detection kits and then visualized using a molecular imager with Image Lab software (Bio-Rad, CA, USA). Protein bands were also quantified with Image Lab software (Bio-Rad, CA, USA). Equal loading of proteins was verified by β-actin (#A5441, Sigma, USA) and GAPDH (AF7021, Affinity Biosciences, USA) immunoblot analysis. At least three separate experiments were performed with different lysates to confirm the changes in protein levels. List of effective concentrations of primary antibodies in Western blotting see Supplementary Figure S2.

#### ELISA

The levels of Aβ40 and Aβ42 were detected using ELISA kits by technicians who were blinded to the experimental groups. For the detection of Aβ40 and Aβ42 levels in cells, the cells were washed, trypsinized, and lysed in extraction buffer (1% CHAPS in TBS, pH 7.6). Intracellular Aβ40 and Aβ42 were then detected. The volume of the medium used was adjusted to the protein concentrations measured in total cell lysates. For the detection of soluble or insoluble Aβ40 and Aβ42 in the brains of WT and APP/PS1 mice, dissected tissue was homogenized in 5 volumes of extraction buffer (1% CHAPS in TBS, pH 7.6). Then, all mixtures were placed on ice for at least 3 h. The homogenates were centrifuged at 70,000 rpm for 20 min at 4°C, and the supernatants were diluted with EIA buffer contained in the kit for the analysis of soluble Aβ40 and Aβ42.

#### Statistical Analysis

All data are expressed as the mean ± standard error of the mean (SEM). Data were analyzed by GraphPad Prism 5 (GraphPad Software, Inc., La Jolla, CA, USA). For the examination of statistically significant differences between two groups, a two-sided, unpaired Student’s *t*-test was used, and for multiple comparisons, a one-way ANOVA followed by the Newman–Keuls test was used. In the Barnes maze training, statistical analysis was performed using a two-way ANOVA followed by the Newman–Keuls test and one-way analysis of variance/Newman–Keuls test for the probe test. The main effects are presented only when the higher second-order interaction was not significant. Values of *p* < 0.05, *p* < 0.01, and *p* < 0.001 were considered statistically significant.

## Results

### Oral DHA Alleviated Memory and Cognitive Deficits in APP/PS1 Double Transgenic Mice

**Figure 2:**
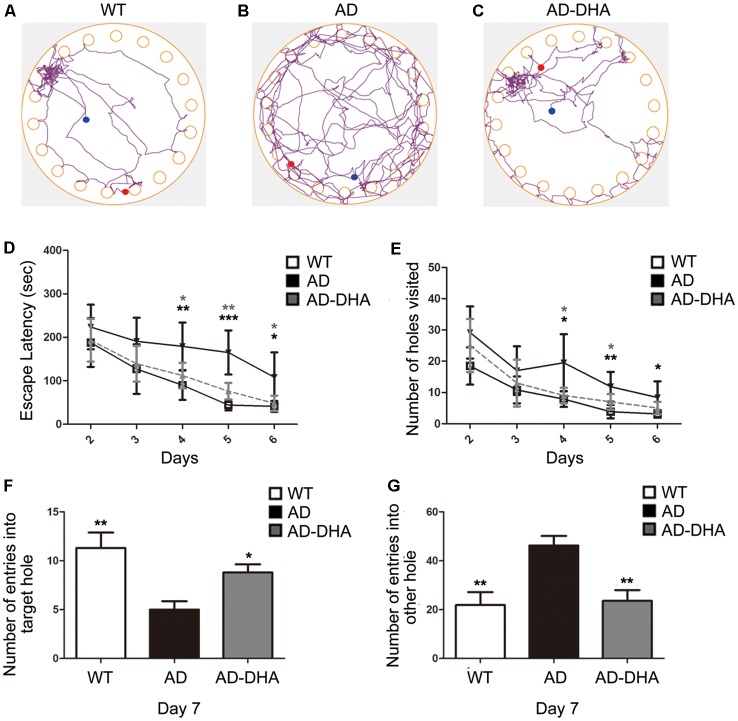
Effect of DHA on APP/PS1 mice in the Barnes maze. Moving tracks of the CTRL group **(A)**, Alzheimer’s disease (AD) group **(B)** and AD-DHA group mice **(C)**. Difference analysis of latency to find the escape box **(D)** and number of wrong holes visited **(E)** on training days, the number of entries into the target holes **(F)** and the other holes **(G)** on the test day in the Barnes maze test. Data are reported as the mean ± standard error of the mean (SEM) of ten animals per group. *p < 0.05, **p < 0.01, and ***p < 0.001 compared to the wild type (WT) group (Two-way ANOVA followed by the Newman–Keuls test for the training test and one-way analysis of variance/Newman–Keuls test for the probe test).

After 3 months of treatment, the mice began the Barnes test, which evaluates spatial learning and memory. After the adaptive training on the first day, the latency to find the escape box and the number of wrong holes visited revealed the main effects of the next 5 days of training for the mice. Mice moving tracks in the CTRL group (Figure 2A), the AD group (Figure 2B), the AD-DHA group (Figure 2C) see in Figure 2. *Post hoc* comparisons showed that on the second (ANOVA: *F*_(3,20)_ = 0.6152, *p* = 0.8610) and third days (*F*_(3,20)_ = 0.7752, *p* = 0.4654), no change in the latency to find the escape box was observed between the groups. Moreover, no significance was noted in the number of wrong holes visited on the second (*F*_(3,20)_ = 2.103, *p* = 0.1337) or the third day (*F*_(3,20)_ = 0.8793, *p* = 0.4213). On the fourth, fifth and sixth days of training, DMSO-treated AD mice had significantly increased latency to find the escape box (+99.64%, 274.29%, and 163.59%, respectively; Figure 2D) and the number of wrong holes visited (+147.78%, 207.53%, and 166.37%, respectively; Figure 2E) when compared to WT mice. Treatment with DHA for AD mice significantly protected against these increases on the fourth, fifth and sixth days of training (−37.20%, 53.94%, and 55.23%, respectively; Figure 2D; −52.41%, 40.43% and, 40.56%, respectively; Figure 2E), when compared to those of the DMSO-treated AD mice.

In the probe test, one-way ANOVA followed by a Newman–Keuls *post hoc* test demonstrated that DMSO-treated AD mice had a decrease in the number of entries into the target hole (55.75%) and an increase in the number of entries into other holes (110.96%), when compared with that of WT mice, and DHA significantly enhanced the accuracy of finding escape holes (approximately 76%) and recognizing the wrong holes (48.92%; Figures 2F,G). Mice treated with DHA did not change their behavioral parameters when compared to those of the WT group (Figures 2F,G; *F*_(3,20)_ = 6.003, *p* < 0.01).

### Oral DHA Decreased the Burden of Aβ Aggregations and SP Without the Participation of AD-Related Degradation Enzymes

**Figure 3:**
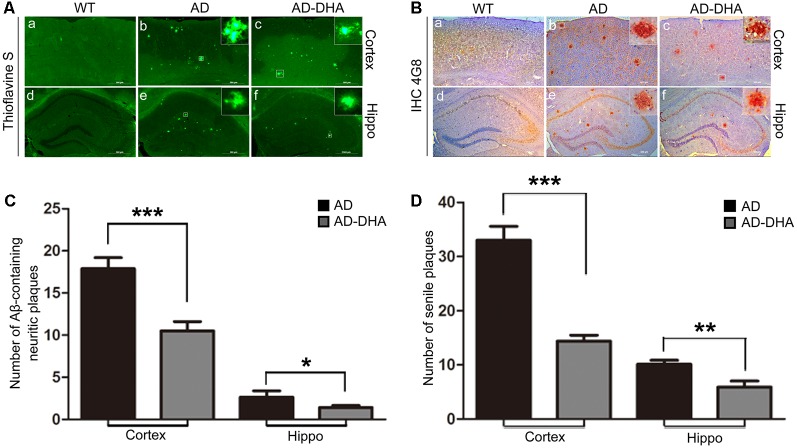
Effects of DHA on Aβ aggregation and senile plaque (SP) deposition. The brains were prepared from mice treated with intragastric administration of DHA (20 mg/kg/day) or DMSO for 90 days. Thioflavin S staining **(Aa–f)** and immunohistochemistry **(Ba–f)** showed Aβ-containing neuritic plaques and SP in the cortex and hippocampus of WT mice, DMSO-treated AD mice, and DHA-treated AD mice. The number of Aβ aggregates **(C)** and the number of SP as determined by immunohistochemistry **(D)** in the cortex and hippocampus are illustrated as histograms. *n* = 6. *p < 0.05, **p < 0.01, and ***p < 0.001 compared to the DMSO-treated AD group. Student’s unpaired *t*-test was performed for histological staining data and ELISA results. Scale bar = 500 μm.

Three mice per group were sacrificed after the behavioral tests to detect Aβ deposition by Thioflavin S staining (Figures 3A,C) and SP by immunohistochemistry (Figures 3B,D) in the cortex and hippocampus, respectively. Thioflavin S staining confirmed that the Aβ burden of both the cortex and hippocampus in the DHA-treated group were significantly lower than those in the DMSO-treated AD group (Newman–Keuls test; *p* < 0.001 in the cortex; *p* < 0.05 in the hippocampus). Anti-Aβ 4G8 antibody staining for immunochemistry (*p* < 0.001 in the cortex; *p* < 0.01 in the hippocampus) also showed that the quantities of SP in the cortex and hippocampus of the DHA-treated group were markedly reduced, which was consistent with the results of the Thioflavin S staining.

**Figure 4:**
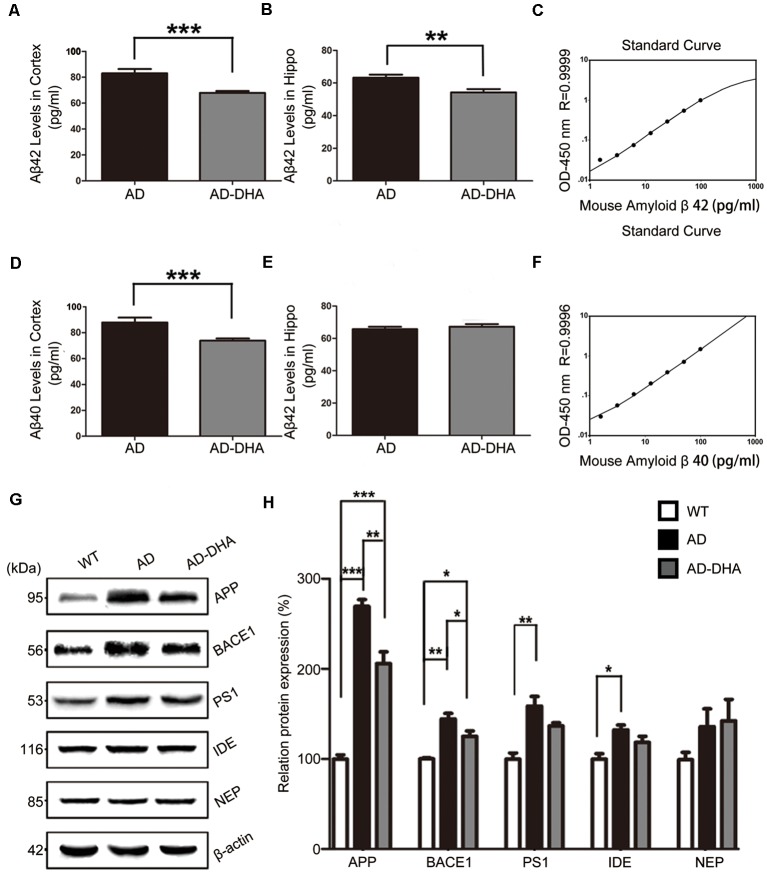
Effects of DHA on eliminating Aβ40, Aβ42, APP, and β-site APP cleaving enzyme (BACE1). Protein levels of Aβ40 and Aβ42 in the hippocampus and cortex were detected by ELISA **(A–F)**. **p < 0.01 and ***p < 0.001 compared to the DMSO-treated AD group. Protein levels of APP, BACE1, PS1, insulin-degrading enzyme (IDE) and NEP in extracts of whole brain tissue were detected by Western blotting (WB), shown in panel **(G)**. Quantification of the WB results are shown in panel **(H)**, and the results are shown as the mean values ± SD. *p < 0.05, **p < 0.01, and ***p < 0.001 between two groups. One-way analysis of variance/Newman–Keuls test was performed for all WB data.

The Aβ40 and Aβ42 levels in the cortex and hippocampus of AD mice (Figures 4A–F). An ELISA assay revealed that DHA markedly reduced Aβ42 production in both the cortex and hippocampus (Newman–Keuls test; *P* < 0.05 in the cortex; *p* < 0.01 in the hippocampus, Figures 4A,B). The standard curve of mouse Aβ42 ELISA assay was shown in Figure 4C. Relative to the AD CTRL group, the burden of Aβ40 in the cortex was markedly downregulated (*p* < 0.001; Figures 4D,E) by oral DHA, while no significant change was found in the hippocampus of the DHA-treated group (*P* > 0.05). The standard curve of mouse Aβ40 ELISA assay was shown in Figure 4F.

Compared with the WT group, the expression of APP in the DMSO-treated AD group was significantly increased (Newman–Keuls test; *p* < 0.001, Figures 4G,H), whereas the treatment with DHA protected against the increase (+169.39% compared to WT, *p* < 0.001; +23.61% compared to AD, *p* < 0.01; Figures 4G,H). Significant differences in the expression of BACE1 were noted between the AD groups (*p* < 0.05, Figures 4G,H). There were no significant differences in the expression of PS1 in the AD groups (*p* > 0.05, Figures 4G,H) after oral DHA treatment. There were no significant differences in the expression of IDE (*p* > 0.05, Figures 4G,H) or NEP (*p* > 0.05, Figures 4G,H) between the AD group and the AD-DHA group, although significant difference was noted in the protein expression of IDE between the WT group and the AD-DHA group (*p* < 0.05, Figures 4G,H).

### Oral DHA Corrected Autophagy Dysfunction in AD Mice Model

**Figure 5:**
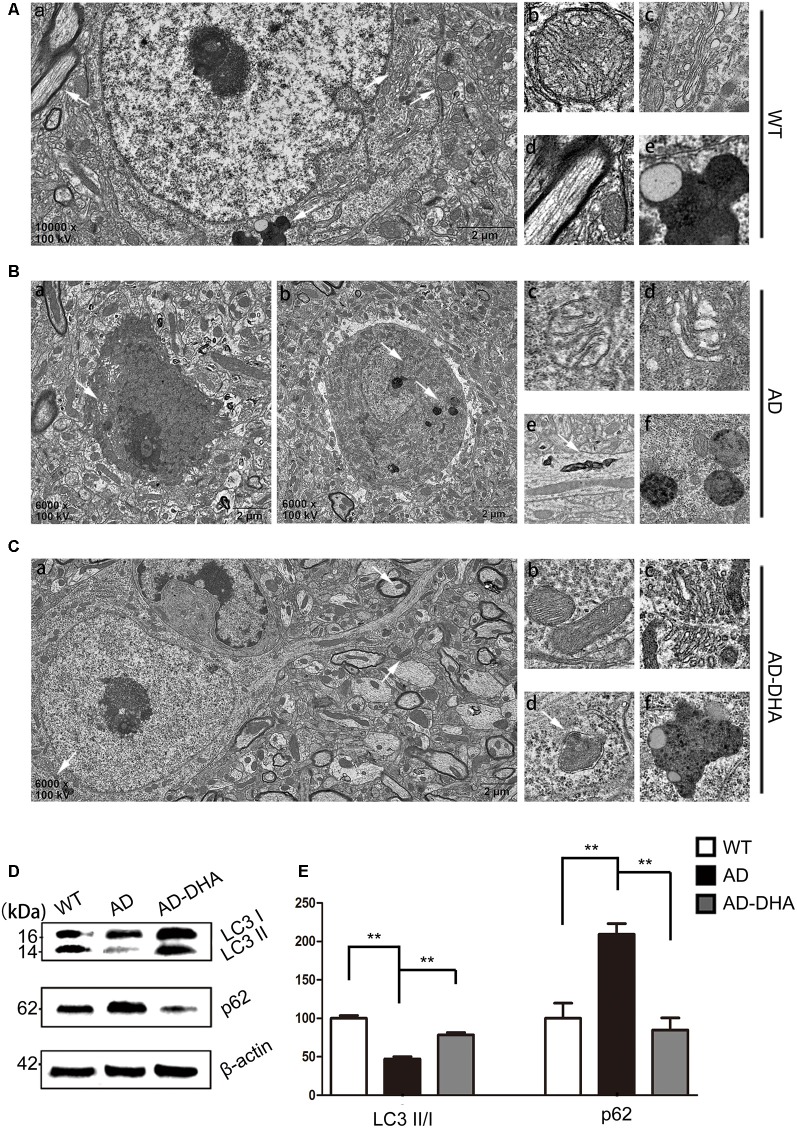
Oral DHA protects the ultrastructures of hippocampal neurons and normalizes autophagic flux. **(Aa,Ba,b,Ca)** Low magnification images of CA1 hippocampal neurons in the three groups were detected by transmission electron microscopy (TEM). Normal mitochondria **(Ab)**, Golgi apparatus **(Ac)**, microtubules **(Ad)**, and granules of lipofuscin with lipid droplets **(Ae)** in the cytoplasm of neurons of WT mice (white arrows in **Aa**). Abnormal mitochondria **(Bc)**, Golgi fragments **(Bd)**, aberrant myelin figures **(Be)**, and late autophagosome deposits **(Bf)** in the neurons of the DMSO-treated AD group (white arrows in **Ba**). Intact mitochondria **(Cb)**, Golgi apparatus **(Cc)**, early autophagosomes **(Cd)**, and granules of lipofuscin with lipid droplets **(Cf)** in the cytoplasm of neurons of DHA-treated mice (white arrows in **Ca**). *Scale bars* **(Aa,Ba,b,Ca)**: 2 μm and **(Ab–e,Bc–f,Cb–f)**: 350 nm. Levels of LC3, p62, and β-actin in extracts of whole brain tissue were detected by WB **(D)**, and quantification of the results is shown in panel **(E)**. **p < 0.01 between two groups. One-way analysis of variance/Newman–Keuls test was performed for all WB data.

In contrast with DMSO-treated AD mice, neuronal autophagy was markedly induced, and the AD-like pathological characteristics were evidently ameliorated in DHA-treated APP/PS1 mice, and there was no significant difference between DMSO-treated WT mice and DHA-treated AD mice (Figures 5A–C). In the CA1 hippocampus in DHA-treated APP/PS1 mice, neurons remained clear and intact (Figure 5Aa). Conversely, dark neurons (DNS; Figure 5Ba) and neurons with autophagic dysfunction (Figure 5Bb) emerged in the CA1 hippocampus of DMSO-treated APP/PS1 mice but did not emerge in the other two groups (Figures 5Aa,Ca). Membrane structures of mitochondria (Figure 5Ab) and Golgi apparatus (Figure 5Ac) were integral, cytoplasm in axons was clear (Figure 5Ad) and lipofuscins emerged (Figure 5Ae) in the CTRL group. In the subcellular ultrastructure of DMSO-treated AD mice, the edema of mitochondria (Figure 5Bc) and hydropic degeneration of the Golgi apparatus (Figure 5Bd) occurred. Axons were filled with aberrant myelin figures that damaged the basic structure of microtubules (Figure 5Be) and late APs were massively deposited around the nucleus (Figure 5Bf). In contrast, organelles such as mitochondria (Figure 5Cb) and Golgi bodies (Figure 5Cc), with the presence of APs with a double membrane that engulfed abnormal organelles (Figure 5Cd) and some granules of lipofuscin present (Figure 5Cf), maintained basically complete organelle structures in DHA-treated AD mice (Figure 5C) and WT mice (Figure 5A).

The ratio of LC3 II/I and the expression level of SQSTM/p62 were used as one of the measures of autophagic flux. The ratio of LC3 II/I was significantly upregulated and the degradation of SQSTM/p62 was significantly hampered in the DMSO-treated AD group, compared with the CTRL group (*p* < 0.01; Figures 5D,E). Oral DHA treatment increased the ratio of LC3 II/I (+65.98% AD vs. AD-DHA, *p* < 0.01; Figures 5D,E) and the blocked degradation of SQSTM/p62 (*p* < 0.01; Figures 5D,E) was significantly reverted in DHA-treated AD group, compared with the DMSO-treated AD group.

**Figure 6:**
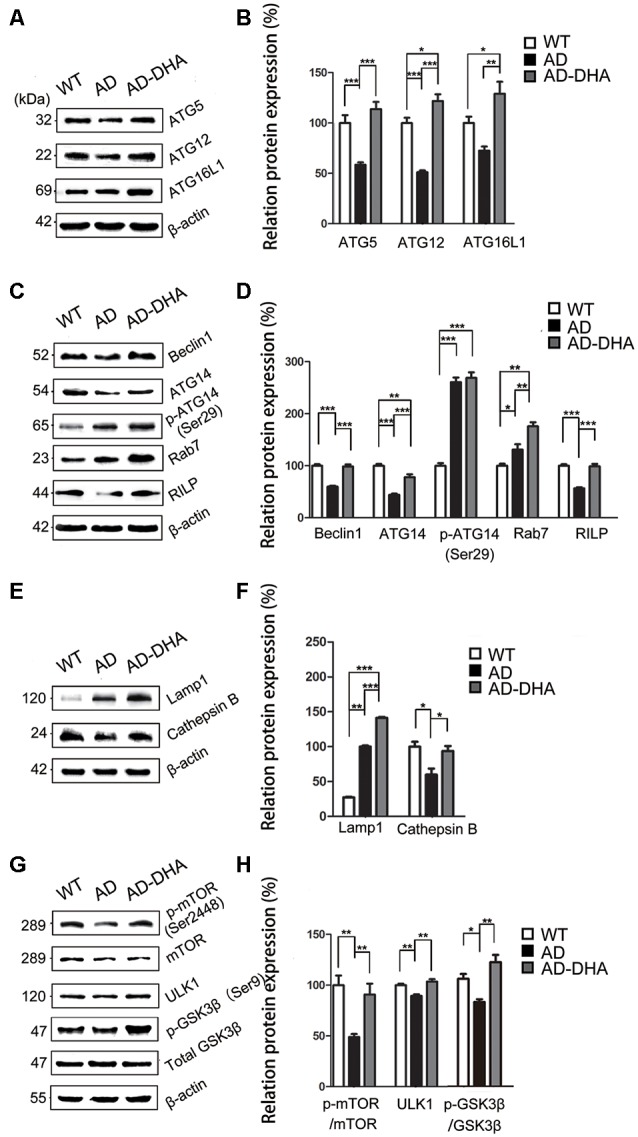
DHA increases the basal level of autophagy. Protein levels of ATG5, ATG12, ATG16L1, Beclin1, ATG14, p-ATG14 (Ser29), Rab7, RILP, Lamp1, CTSB, p-mTOR(Ser2448), mammalian target of rapamycin (mTOR), ULK1, p-GSK3β (Ser9), GSK3β in extracts of whole brain tissue were detected by WB. Representative Western blots are presented in panels **(A,C,E,G)**, and quantification of the results is shown as the mean values ± SD in panels **(B,D,F,H)**. *p < 0.05, **p < 0.01, and ***p < 0.001 between two groups. One-way analysis of variance/Newman–Keuls test was performed for all WB data.

Expression of ATG5 (*p* < 0.001; Figures 6A,B) and ATG12 (*p* < 0.001; Figures 6A,B) were markedly decreased in the AD group compared with that of the WT group, which were protected by DHA treatment. Although there was no significant difference in the level of ATG16L1 between the WT and AD CTRL groups (*p* > 0.05), DHA treatment enhanced the expression of ATG16L1 in AD mice (*p* < 0.01; Figures 6A,B).

Expression of Beclin1 (*p* < 0.001; Figures 6C,D) and ATG14 (*p* < 0.01; Figures 6C,D) were markedly decreased in the AD group compared with that of the WT group, which were protected by DHA treatment. Conversely, no significant difference in p-ATG14 (Ser29) between the AD groups was noted (*p* > 0.05; Figures 6C,D). The levels of Rab7 (*p* < 0.05; Figures 6C,D) were significantly upregulated and RILP (*p* < 0.001; Figures 6C,D) was downregulated by overexpression of APP/PS1 in the AD model, compared with that of the WT mice. As shown in Figures 6C,D, the effects of oral DHA upregulated the expression level of Rab7 (*p* < 0.01; Figures 6C,D) and RILP (*p* < 0.001; Figures 6C,D), compared with that of the DMSO-treated AD group.

The levels of Lamp1 (*p* < 0.01; Figures 6E,F) were significantly upregulated by overexpression of APP/PS1 in the AD model, while the expression of CTSB (*p* < 0.05; Figures 6E,F) was inhibited compared with that of the WT mice. Rather, as shown in Figures 6E,F, the effects of oral DHA significantly upregulated the levels of Lamp1 (*p* < 0.001), and CTSB (*p* < 0.05; Figures 6E,F).

The ratio of expression of phosphorylated/total mTOR was used as a measure for autophagy inhibition. Oral DHA treatment increased the p/t mTOR ratio (Newman–Keuls test; +100.81% AD vs. AD-DHA, *p* < 0.01; Figures 6G,H), suggesting rejuvenation in autophagic activity in AD mice. Expression of ULK1 (*p* < 0.01; Figures 6G,H) was markedly decreased in the AD group compared with that of the WT group, which were protected by DHA treatment. The ratio of expression of phosphorylated/total GSK3β was used as an indirect measure of GSK3β activity. Oral DHA treatment increased the p/t GSK3β ratio (+approximately 47.28% AD vs. AD-DHA, *p* < 0.01; Figures 6G,H), suggesting decreased the enzyme activity of GSK3β in AD mice. The values of total and phosphorylated GSK3β are presented in Figures 6G,H.

### Treatment With DHA Had Multitarget-Regulating Effects on Autophagy Flux in AD Model Cells

**Figure 7:**
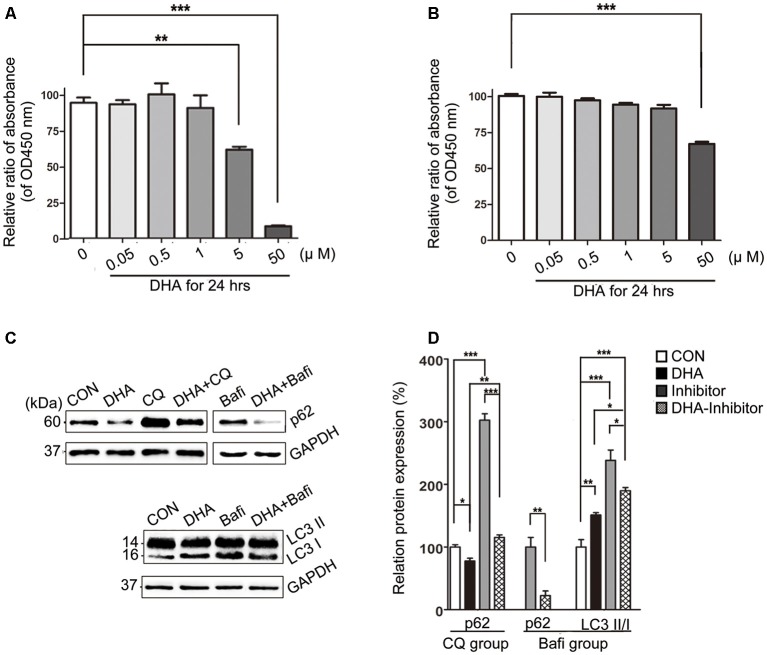
DHA ameliorates the protein levels of autophagic fusion and degradation processes. **(A,B)** DHA reduced the cell viability in a dose-dependent manner both in N2a-APP cells and APP-SH-SY5Y cells. The data are presented as the means ± SEM and were analyzed by one-way ANOVA (Newman–Keuls test, **p < 0.01, ***p < 0.001). **(C)** Protein levels of SQSTM/p62, LC3B I and LC3 II in cells in the CON group, DHA group, chloroquine (CQ) group, CQ-DHA group, Bafi group and Bafi-DHA group were detected by WB. Representative Western blots are presented in panels **(C)**, and quantification of the results is shown as the mean values ± SD in panels **(D)**. *p < 0.05, **p < 0.01, and ***p < 0.001 between two groups. One-way analysis of variance/Newman–Keuls test was performed for all WB data.

The cell viability was significantly inhibited in N2a-APP with 5 μM or 50 μM of DHA and APP-SH-SY5Y cells treated with 50 μM of DHA in 24 h. The relative rate was calculated using the following formula: each group/Control group × 100% (Figures 7A,B).

WB analysis of N2a-APP cells revealed a marked difference in the autophagic flux marker proteins, LC3 and p62, comparing levels in the CON group and DHA group with that of the CQ group, Bafi group, Bafi-DHA group, and CQ-DHA group (Figures 7C,D).

**Figure 8:**
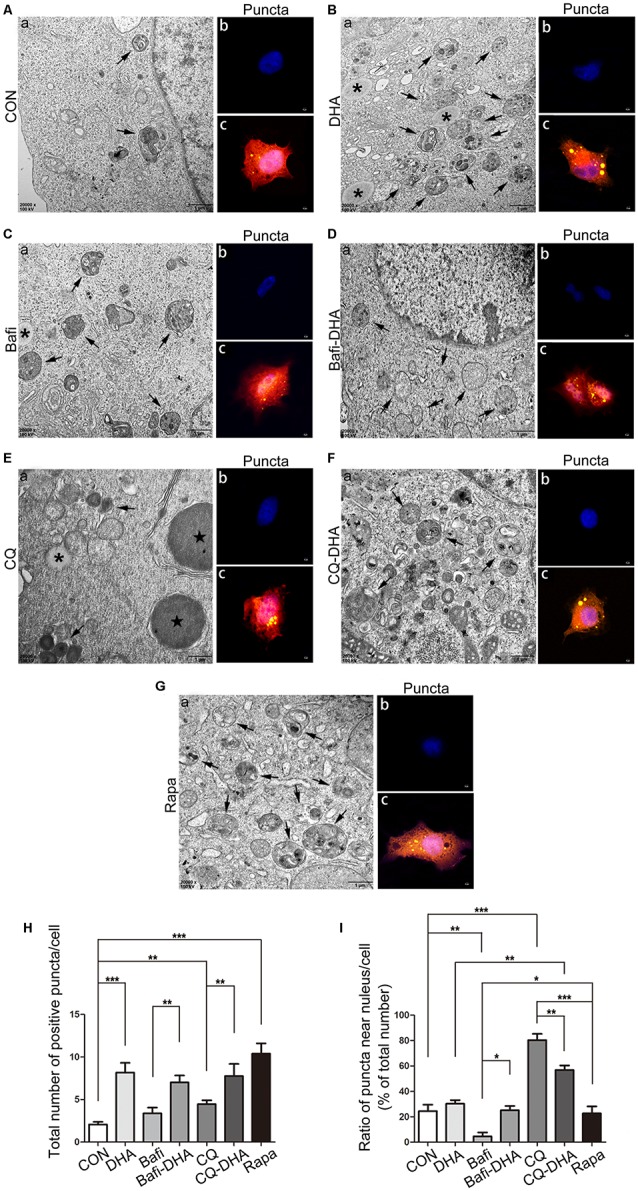
DHA stimulated autophagosomes (APs) formation and affected autophagic flux morphology. **(Aa**,**Ba**,**Ca**,**Da**,**Ea**,**Fa**,**Ga)** High magnification images of APs (arrows in **Aa**,**Ba**,**Ca**,**Da**,**Ea**,**Fa**,**Ga**) and lysosomes (*Aa,**Ba**,**Ca**,**Da**,**Ea**,**Fa**,**Ga**) or lipid droplets (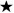 in **Aa**,**Ba**,**Ca**,**Da**,**Ea**,**Fa**,**Ga**) of N2a-APP cells in the seven groups were detected by TEM after treatment with DMSO **(Aa)**, DHA (1 μM, 24 h) **(Ba)**, bafilomycin A1(50 nM, 25 h) **(Ca)**, bafilomycin A1 (50 nM, 25 h) and DHA (1 μM, 24 h) **(Da)**, CQ (25 μM, 25 h) **(Ea)**, CQ (25 μM, 25 h) and DHA (1 μM, 24 h) **(Fa)**, rapamycin (100 nM, 24 h) **(Ga)**. **(Ab,c**,**Bb,c**,**Cb,c**,**Db,c**,**Eb,c**,**Fb,c**,**Gb,c)** APP-SH-SY5Y cells were transfected with a tandem mRFP-GFP-LC3 adenovirus for 24 h, followed by treatment with DHA (1 μM, 24 h) **(Bb,c)**, bafilomycin A1 (1 nM, 25 h) **(Cb,c)**, bafilomycin A1 (1 nM, 25 h) and DHA (1 μM, 24 h) **(Db,c)**, CQ (10 μM, 25 h) **(Eb,c)**, CQ (10 μM, 25 h) and DHA (1 μM, 24 h) **(Fb,c)** and rapamycin (100 nM, 24 h) **(Gb,c)**. APs were visualized in each group as yellow puncta under a confocal microscope. Scale bar (**Aa**,**Ba**,**Ca**,**Da**,**Ea**,**Fa,Ga)**: 1 μm and **(Ab,c,Bb,c,Cb-c,Db,c,Eb,c,Fb,c,Gb,c)**: 5 μm. **(H,I)** Positive puncta were counted, and the ratio of puncta near the nucleus in each cell was calculated. Randomly selected fields were counted, and the data are presented as the means ± SEM and were analyzed by one-way ANOVA (Newman–Keuls test, *p < 0.05, **p < 0.01, and ***p < 0.001 compared to CON group).

TEM analysis of N2a-APP cells and fluorescence microscopy analysis of APP-SH-SY5Y cells transfected with mRFP-GFP-LC3 adenoviruses showed a significant difference in key biomarkers of autophagy flux, APs, and lysosomes, comparing levels in the CON group and DHA-treated (DHA) group with that of the CQ-treated group, bafilomycin A1-treated (Bafi), Bafi-DHA-treated (Bafi-DHA) group, CQ-DHA-treated (CQ-DHA) group and positive CTRL group, which was the rapamycin (Rapa) group (Figure 8).

DHA treatment markedly increased the number of positive puncta (Newman–Keuls test; +295.2% CON vs. DHA, *p* < 0.001; Figures 8Ab,c for the CON group and Bb-c for the DHA group; Figure 8H for the histogram), suggesting an increase in the autophagic activity of APP-SH-SY5Y cells, which is in accordance with the TEM data comparing the CON group and DHA group (Figure 8Aa for the CON group and Ba for the DHA group; Figure 8I for the histogram). Moreover, the ratio of LC3 II/I increased (+51.0% CON vs. DHA, *p* < 0.01; Figures 7C,D) and the level of p62 decreased (−22.4% CON vs. DHA, *p* < 0.05; Figures 7C,D) with DHA treatment of N2a-APP cells, which is consistent with results comparing the AD mice with the AD-DHA mice groups (Figures 7C,D).

Bafilomycin A1 treatment significantly downregulated the ratio of positive puncta near the nucleus (Newman–Keuls test; −81.14% CON vs. Bafi, *p* < 0.01; Figures 8Ab,c for the CON group and Cb-c for the Bafi group; Figure 8I for the histogram), suggesting that the fusion between APs and lysosomes near the nucleus was impaired, and the increased number of positive puncta in the Bafi group compared with that of the CON group was not significantly different (*p* > 0.05; Figures 8Ab,c,Cb,c,I for the histogram). APs dyed dark gray, suggest that late APs accumulated in the cytoplasm after bafilomycin A1 treatment (Figures 8Ca–c). TEM results coincided with the results shown in Figures 7C,D that the ratio of LC3 II/I significantly increased when comparing the Bafi group with that of the CON group (+138.2% CON vs. Bafi, *p* < 0.001; Figures 7C,D). Not only the number of positive puncta (+107.4% Bafi vs. Bafi-DHA, *p* < 0.01; Figures 8Cb,c,H) but also the ratio of puncta near the nucleus markedly increased (+447.9% Bafi vs. Bafi-DHA, *p* < 0.05; Figures 8Cb,c,Db,c,I) in the Bafi-DHA group compared with that of the Bafi group. Furthermore, WB analysis showed that DHA treatment ameliorated the increase in the ratio of LC3 II/I induced by bafilomycin A1 (*p* < 0.05; Figures 7C,D) and downregulated the level of p62 in the Bafi-DHA group compared with that of the Bafi group (*p* < 0.01; Figures 7C,D).

CQ hindered APs degradation by lysosomes, which was manifested by the increased number of APs (Newman–Keuls test; +130.8% CON vs. CQ, *p* < 0.01; Figures 8Eb,c,H), the increased ratio of positive puncta near the nucleus (Newman–Keuls test; +224.6% CON vs. CQ, *p* < 0.001; Figures 8Eb,c,I) and upregulation of p62 (Newman–Keuls test; +202.4% CON vs. CQ, *p* < 0.001; Figures 7C,D) compared with those of the CON group. Additionally, large lysosomes and APs dyed dark gray aggregated in the cytoplasm after CQ treatment (Figure 8Ea). Fluorescence microscopy analysis showed that DHA treatment not only further enhanced the production of APs in the CQ+DHA group (Newman–Keuls test; +67.44% CQ vs. CQ+DHA, *p* < 0.05; Figures 8Eb,c,Fb,c,H) but also increased the ratio of positive puncta away from the nucleus by 27.86% (*p* < 0.01; Figure 8I) compared with that of the CQ group, although the ratio was smaller than that of the DHA group (Newman–Keuls test; +87.97% DHA vs. CQ+DHA, *p* < 0.01; Figures 8Bb,c,Fb,c,I). WB data showed that DHA rescued the degradation dysfunction of p62 caused by CQ (Newman–Keuls test; −187.1% CQ vs. CQ+DHA, *p* < 0.001; Figures 7C,D). No large lysosomes were detected in the CQ+DHA group (Figure 8Fa).

## Discussion

The brain neuropathology of AD is characterized by synaptic abnormalities and neuronal degeneration, as well as extracellular Aβ deposition and intraneuronal neurofibrillary tangles, leading to a decline in memory and other cognitive functions (Ho et al., 2014). Recent studies have suggested that the molecular level of autophagic maintenance might be a suitable way to treat AD as well as some other neurodegenerative diseases, since it could not only prevent cell death but also effectively degrade pathological proteins, such as Aβ, p-Tau, α-synuclein, and glutamine repeats (Ghavami et al., 2014). Autophagy, as a lysosomal mediated process of cell self-processing, is closely related to the clearance of abnormal accumulation of AD-related proteins. However, a large number of studies have reported that there are dual roles for autophagy, which are degradation and secretion of AD Aβ peptide (Choi et al., 2018; Uddin et al., 2018). There are a large number of APs and autolysosomes (ALs) in the brain of AD patients, which are caused by the activation of autophagy on the one hand, the fusion obstacle between APs and lysosome, or the degradation reduction of ALs on the other hand. In the early stage of AD, autophagy can accelerate the clearance of denatured protein and promote the survival of neurons. With the development of AD, the aggregation of APs is increased and the clearance of ALs decreased. Therefore, it is of great scientific significance to study the dynamic changes of autophagy flux in the pathogenesis of AD and to find new targets for the prevention and treatment of AD.

DHA, the active metabolite of artemisinin, may meet the conditions for the induction of basal autophagy and correction of autophagic flux (Ho et al., 2014; Efferth, 2017; Lam et al., 2018). However, no study has mentioned the therapeutic effect of DHA in AD regarding the degradation of toxic aggregated proteins by inducing autophagy. Thus, it is vital to determine the potential effects and mechanisms of DHA treatment on AD. In the present study, Barnes maze test results showed that DHA administration by gastric perfusion for 3 months exhibited a protective effect on learning and memory impairment in the APP/PS1 double transgenic mice. Then we found that the diameters of SP were smaller and the densities of SP were lower in the DHA-treated group compared with those of the DMSO-treated group, which supports our assumption that DHA may prevent the aggregation of Aβ and deposition of SP. The main forms of Aβ in the brain are Aβ40 and Aβ42, the latter is more prone to amyloidosis and toxicity. To verify the potential effects of DHA on the levels of Aβ40 and Aβ42 in the cortex and hippocampus, ELISA results showed that the levels of Aβ42 in both the cortex and hippocampus and of Aβ40 in the cortex were markedly reduced by oral DHA, which indicates that DHA may normalize the burden of Aβ42 that acts as more harmful peptides prone to misfolding and aggregating, but not Aβ40, and plays a central role in the pathological course of AD (Querfurth and LaFerla, 2010; Lane et al., 2018). In addition, hippocampal neurons are more prone to protein aggregation than neurons in the cortex, which may suggest that this selective difference is closely related to the degradation rate of aggregated proteins; the degradation rate is based on cell- and region-specific autophagy activity and vulnerability of substrates to clearance *via* autophagy, possibly due to neuronal dependency on autophagic flux (Lumkwana et al., 2017). We found that the anti-Aβ42 effect of DHA on the cortex was more significant than that on the hippocampus, as well as the anti-Aβ40 effect of DHA on the cortex. Consequently, we considered that DHA exhibited neuroprotective effects in AD. The imbalance of Aβ metabolism leads to the formation of extracellular SPs and a series of pathological changes. Aβ is formed by the cleavage of APP protein by β- and γ-secretase, and BACE1 and PS1 are the active centers of β- and γ-secretases, respectively. In this study, the results showed that compared with the control group, the levels of APP and BACE1 protein were down-regulated after DHA treatment, indicating that Aβ production was reduced. Then we tested the degradation of Aβ. The degradation of extracellular Aβ is mainly completed by IDE and NEP (Yamamoto et al., 2018). However, our results showed that DHA treatment did not significantly change the levels of IDE and NEP proteins compared with the control group. While the degradation of intracellular Aβ is mainly transported to lysosome through endocytosis or autophagy, there is increasing evidence that autophagy dysfunction accompanies the development of AD (Choi et al., 2018; Uddin et al., 2018). Dysfunction in the autophagy-lysosome pathway is closely related to the production, clearance and neurotoxicity of Aβ. In the current study, TEM results showed that there were a large number of normal neurons in the brain of DHA-treated mice as the WT mice. In addition, double-membraned APs enveloped organelles and cytosolic proteins instead of aberrant myelin accumulation occurred in DHA-treated neurons. At the same time, no DNs and neurons with autophagic dysfunction could be found in DHA-treated neurons. Therefore we speculate that autophagy could be markedly improved by DHA treatment in an AD mouse model. The levels of autophagy-associated proteins were assessed by WB to verify the hypothesis that oral DHA could protect against AD pathophysiology by inducing autophagy in AD-like neurons. We found that the level of SQSRM1/p62 increased and the ratio of LC3 II/I decreased in AD brains compared with that of WT brains, which indicates low autophagic activity and obstructed autophagic flux in AD pathogenesis (Boland et al., 2008). The relatively low level of LC3 II along with a high level of Aβ burden in AD mice brain might be explained by the lack of a potential protective role for LC3-associated endocytosis that downregulates the levels of neurotoxic Aβ (Heckmann et al., 2019).

We next investigated the mechanism by which DHA activates autophagy. The first step is autophagy initiation and APs formation. The levels of proteins associated with isolation of AP membranes from the ER, such as ULK1 and Atg14, proteins associated with the elongation of AP membranes, such as ATG12, ATG5, ATG16L1, and LC3. The second step is AL formation (via the fusion of APs and lysosomes), proteins associated with the ALs fusion, such as ATG14, p-ATG14, Rab7, and RILP. The third step is AL degradation: autophagy-encapsulated proteins are released by CTSB and CTSD degradation, and lysosomal acidification plays an important role in their degradation. The WB results showed that the expression of proteins involved in the early stage of autophagy, such as ATG5, ATG12 and ATG16L, were upregulated in DHA treated group, indicating that autophagy was activated. At the middle stage of autophagy, the levels of LC3 II/I were increased, and the p62 protein level was decreased, indicating that autophagy was induced by DHA treatment. ATG14 has been shown to form a complex with Beclin1 in the early stage of autophagy and promote the fusion of APs and lysosomes in the middle stage of autophagy (Diao et al., 2015). However, after treatment with DHA, the levels of Beclin1, ATG14, Rab7 and RILP proteins were significantly increased, indicating that DHA treatment not only activated autophagy but also promoted the fusion of autophagy and lysosome. Studies have shown that reducing the content of proteases in lysosomes and inhibiting the activity of lysosomal enzymes can lead to the accumulation of Aβ in cells. In addition, the endocytosis of Aβ can, in turn, reduce the function of lysosomes and affect the degradation of Aβ by lysosomes (Zheng et al., 2012; Lauritzen et al., 2016). The results of this study showed that the protein expression of the lysosomal protease CTSB and the lysosomal marker Lamp1 in the DHA-treated mice increased compared with that of the CRTL group, suggesting that DHA treatment can also increase the number of lysosomes and their degradation function.

GSK3β-TIP60-ULK1 pathway and mTOR/ ULK1 pathway are two classic autophagy activation pathways (Nie et al., 2016). In this study, WB showed that DHA inhibited the activity of mTOR, then activated ULK, indicating that DHA can also activate autophagy by inhibiting the mTOR/ ULK1 signaling pathway, but DHA does not activate autophagy by activating GSK3.

**Figure 9:**
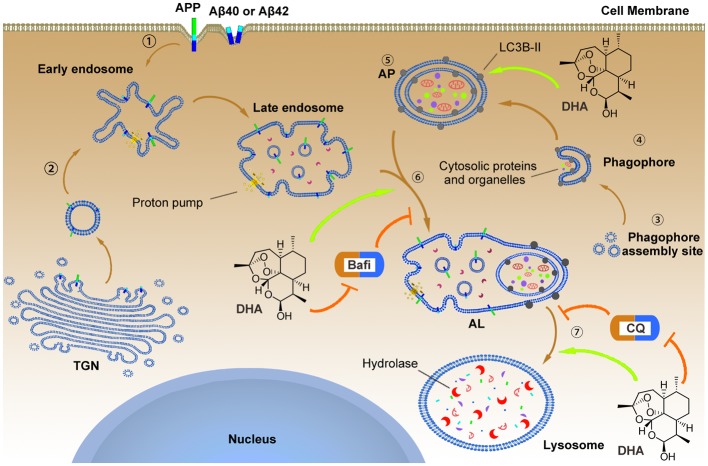
The underlying mechanism by which DHA improves autophagic flux and clears Aβ40 and Aβ42. 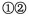 APP, Aβ40 and Aβ42 can reach the early endosome either *via* the trans-Golgi network (TGN) or *via* the cell surface. 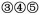 In parallel, normal autophagy is induced by the formation of APs that initiate at the phagophore assembly site; nucleation of the phagophore, sequestration of substrates, and expansion of the double membrane are involved in this process, which is markedly hindered in AD pathology. To reverse this process, DHA upregulated the levels of the Atg5-Atg12-Atg16L complex to facilitate LC3 lipidation (LC3 I) with phosphatidylethanolamine that was inhibited by AD-like pathogenesis 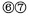. In addition, the treatment of DHA protected against the bafilomycin A1-induced abnormal fusion of APs and late endosomes, ameliorated the CQ-mediated loss of lysosomal degradation function, and finally promoted the degradation of Aβ40 and Aβ42.

To clarify the potential mechanisms by which DHA affects autophagic flux, N2a-APP cells and APP-SH-SY5Y cells were treated with DHA, followed by treatment with bafilomycin A1, an autophagic fusion agonist, CQ, and autophagic degradation agonist, and rapamycin, an autophagic initiation antagonist. We found that DHA indeed accelerated the degradation of p62 in the cell model compared with that of the CON group. In addition, CQ treatment significantly hampered the degradation of p62 in the cell model, which was consistent with its inhibition of degradative enzyme activity (Homewood et al., 1972), and was markedly ameliorated by DHA treatment, which reversed the blocked degradation of aggregated proteins and damaged organelles in lysosomes. To detect the process of APs formation mediated by DHA, autophagic agonists and antagonist, TEM and shoots for puncta formation of fluorescent-tagged LC3 proteins in the cytoplasm under fluorescence microscopy were utilized (Klionsky et al., 2012). We found that a large number of lipid droplets and large lysosomes accumulated in the cytoplasm near the nucleus after CQ treatment. Increasing the accumulation of lysosomes indicates that APs and their contents could not be cleared smoothly by lysosomes, which was notably improved in the CQ-DHA group. The phenomenon was also detected by fluorescence microscopy in the CQ group and the CQ-DHA group, in which positive puncta that aggregated near the nucleus were corrected by DHA treatment. Then, we found that the reinforced degradation of p62 by DHA was not disrupted by bafilomycin A1, an autophagic degradation agonist that inhibits the transport of lysosomal protons and leads to blocking the fusion between APs and lysosomes (Klionsky et al., 2008). In addition, DHA not only upregulated the basal level of LC3 II in N2a-APP cells compared with that of the CON group but also reversed the accumulation of LC3 II caused by bafilomycin A1 treatment. Moreover, a mass of stacked APs dyed dark gray (indicating late APs) in the cytoplasm in the Bafi group were mostly replaced by APs dyed light gray (indicating early APs) in the Bafi-DHA group; however, a few late APs were still measured by TEM. The trend was also consistent with the trend in the Bafi group and the Bafi-DHA group under fluorescence microscopy. In addition, differences between the effects of DHA and rapamycin on autophagy mediation and the Aβ clearance are discussed in our study. Unlike rapamycin-induced initiation of autophagy to improve Aβ pathology (Majumder et al., 2011), DHA mainly contributes to maintaining the fusion and AL-digestion stage in autophagic flux as we described above. Moreover, we found that DHA helps clear Aβ42 levels in both the cortex and hippocampus and Aβ40 levels in the cortex of APP/PS1 mice, but rapamycin downregulates Aβ42 but not Aβ40 levels (Spilman et al., 2010), which indicates DHA may have a wider effect for Aβ clearance. Thus, based on the evidence above, we found that DHA treatment can contribute to the clearance of Aβ by maintaining autophagy flux *in vivo* and *in vitro* (Figure 9).

## Conclusion

Overall, DHA treatment can ameliorate behavioral and biochemical deficits in an AD mouse model. The major mechanisms of the therapeutic effects of DHA involve enhancing autophagy, avoiding excessive neuronal death and recovering autophagic flux in AD models *in vivo* and *in vitro*. As AD is undoubtedly caused by multiple factors, and there are many pathological ongoing processes, including a shortage of neurotrophic factors, accumulation of aggregated proteins and progressive neuron loss, one might assume that effective treatment of this disease should include corrections to all these processes. This study demonstrated that DHA can prevent neurons death through raising the overall level of autophagy and maintaining the proper function of autophagy flux in the fusion and degradation stages that predominantly reduces the burden of pathological proteins (such as Aβ40, Aβ42, in this work) in AD models *in vivo* and *in vitro*. Hence, DHA, the active metabolite of *Artemisia annua*, might be a multifunctional molecule capable of correcting the pathology of this disease. Owing to the fact that DHA has been shown to be safe and versatile in many clinical trials worldwide, we suggest that the use of this compound might be considered a therapeutic treatment to reduce and/or prevent symptoms of AD in humans.

## Data Availability Statement

The datasets used and/or analyzed during the current study are available from the corresponding author upon reasonable request.

## Ethics Statement

The animal study was reviewed and approved by the Ethics Committee of Chongqing Medical University.

## Author Contributions

GH, MF, and YZ conceived, designed and supervised the entire study. YZ performed the experiments, acquired data, analyzed data, and drafted the manuscript. YD, TJ, JL, and YLi participated in designing research studies, conducted experiments, acquired data, analyzed data and provided reagents. KW performed TEM, measured autophagic flux *in vivo* and *in vitro*, acquired and analyzed data. YLiu and XP conducted statistical analysis and generated the figures. ZL and GH reviewed and edited the manuscript. All authors have read and approved the final manuscript.

## Conflict of Interest

The authors declare that the research was conducted in the absence of any commercial or financial relationships that could be construed as a potential conflict of interest.

## Abbreviations

Aβ: Amyloid-β peptide
AD: Alzheimer’s disease
AL: Autolysosome
ALs: Autolysosomes
AP: Autophagosome
APP: Amyloid-β protein precursor
APs: Autophagosomes
APS: Ammonium persulfate
BACE1: β-site APP cleaving enzyme
Bafi: Bafilomycin A1
Cathepsin B: CTSB
CQ: Chloroquine
Control: CTRL
DHA: dihydroartemisin
DMSO: Dimethyl sulfoxide
DNs: dark neurons
ECL: Enhanced chemiluminescebce
GSK-3: Glycogen synthase kinase-3
IDE: Insulin degrading enzyme
IHC: Immunohistochemistry
LAMP-1: lysosome associated membrane protein 1
LC3: microtubule-associated protein light chain 3
LTP: long-term potentiation
mTOR: mammalian target of rapamycin
NEP: Neprilysin
PAGE: Polyacrylamide
PBS: Phosphate buffered saline
PBST: PBS with 0.1% Tween-20
PS1: Presenilin 1
PVDF: Polyvinylidene difluride
Rab7: Ras-related protein 7
RAPA: rapamycin
RILP: Rab-interacting lysosomal protein
SDS-PAGE: SDS-polyacrylamide gel
SP: Senile Plaque
Tau: Tau protein
TEM: transmission electron microscopy
TEMED: Tetramethylenediamine
WB: Western blotting
WT: Wild type.
