# Supplementary material for: Dihydroartemisinin Ameliorates Learning and Memory in Alzheimer’s Disease Through Promoting Autophagosome-Lysosome Fusion and Autolysosomal Degradation for Aβ Clearance
Source: Front Aging Neurosci. 2020 Mar 2;12:47. doi: 10.3389/fnagi.2020.00047 (PMC7067048; doi:10.3389/fnagi.2020.00047)
Supplement: Supplementary file 1 [file Image_1.pdf]

Additional files

The drug safety of DHA in mice was reflected by the concentration of the serum GPT/ALT and GOT/AST

Because artemisinin and its derivatives were mainly metabolized in the liver (Gautam A et al., 2009), the safety of DHA in mice for three months was reflected by the concentration of the serum GPT/ALT and GOT/AST B36 (Supplementary Figure S1) and was verified it drug safety. After the three-month treatment with DHA, no obvious adverse reactions in liver function indexes were observed in the mice. Statistical analysis showed that there was a significant difference in GPT/ALT between WT and AD, but there was no remarkable difference in GOT/AST ($P<0.05$) among the WT, AD and AD-DHA groups ($P > 0.05$) during the treatment period.

Indexes	WT group	AD group	AD-DHA group
ALT(U/1)	10.21±4.01	23.92±9.46	14.96±5.57
AST(U/1)	30.13±10.36	41.04±15.54	42.27±7.49

Fig S1. Effect of DHA on liver function of APP/PS1 double transgenic mice during treatment. Statistical analysis showed that there were no significant differences in body weight among the WT, AD and AD-DHA groups during the treatment period ($P > 0.05$).

Reference:

Anirudh Gautam , Tausif Ahmed, Vijay Batra, Jyoti Paliwal. Pharmacokinetics and Pharmacodynamics of Endoperoxide Antimalarials. 2009; 10 (3), 289-306.
