# Supplementary material for: Dihydroartemisinin Ameliorates Learning and Memory in Alzheimer’s Disease Through Promoting Autophagosome-Lysosome Fusion and Autolysosomal Degradation for Aβ Clearance
Source: Front Aging Neurosci. 2020 Mar 2;12:47. doi: 10.3389/fnagi.2020.00047 (PMC7067048; doi:10.3389/fnagi.2020.00047)
Supplement: Supplementary file 2 [file Image_2.pdf]

List of effective concentrations of primary antibodies in Western blot

Antibody Name	Antibody Sourse	Catalog number	Manufacturer	Dilutions
APP	Rabbit mAb	ab32136	Abcam	1/10000
BACE1	Rabbit mAb	ab183612	Abcam	1/1000
PS1	Rabbit mAb	ab76083	Abcam	1/5000
IDE	Rabbit mAb	ab32216	Abcam	1/1000
NEP	Rabbit mAb	ab58968	Abcam	1/1000
GSK3 beta	Mouse mAb	ab93926	Abcam	1/1000
GSK3 beta (phospho S9)	Rabbit mAb	ab75814	Abcam	1/10000
mTOR	Rabbit mAb	AF6308	Affinity	1/1000
mTOR (phospho S2448)	Rabbit mAb	AF3308	Affinity	1/1000
ULK1	Rabbit mAb	DF7588	Affinity	1/500
Beclin 1	Rabbit mAb	ab62557	Abcam	1/1000
ATG14	Rabbit mAb	NBP2- 36445	Novus Bio	1/1000
ATG14 (phospho S29)	Rabbit pAb	AF2320	Affinity	1/1000
ATG5	Rabbit mAb	DF6010	Affinity	1/500
ATG12	Rabbit mAb	DF7937	Affinity	1/1000
ATG16L1	Rabbit mAb	DF3825	Affinity	1/1000
LC3	Rabbit mAb	#3868	CST	1/1000
Rab7	Rabbit mAb	#9367	CST	1/1000
RILP	Rabbit pAb	ab140188	Abcam	1/1000
Lamp1	Rabbit pAb	ab24170	Abcam	1/1000
Cathepsin B	Rabbit mAb	#31718	CST	1/1000
SQSTM1/p62	Rabbit mAb	#5114	CST	1/1000
β-actin	Mouse mAb	#A5441	Sigma	1/5000
GAPDH	Rabbit pAb	AF7021	Affinity	1/3000

* Effective concentrations were observed in their coresponding structions

福昕PDF编辑器
